# The Seduction Script: Psychological and Cultural Norms of Interpersonal Approaches As Markers for Sexual Aggression and Abuse

**DOI:** 10.3389/fpsyg.2016.02070

**Published:** 2017-01-10

**Authors:** Steffen Landgraf, Isabella von Treskow

**Affiliations:** ^1^Clinic of Forensic Psychiatry and Psychotherapy, District Hospital Regensburg, Medizinische Einrichtungen des Bezirks Oberpfalz (Medbo)Regensburg, Germany; ^2^Faculty of Law, University of RegensburgRegensburg, Germany; ^3^Faculty for Linguistics, Literature, and Cultural Science, University of RegensburgRegensburg, Germany

**Keywords:** script, seduction, interpersonal approach behavior, psychology, cultural science, sexual aggression, sexual abuse, nature-nurture debate

## Abstract

Hardly any subjects enjoy greater – public or private – interest than the art of flirtation and seduction. However, interpersonal approach behavior not only paves the way for sexual interaction and reproduction, but it simultaneously integrates non-sexual psychobiological and cultural standards regarding consensus and social norms. In the present paper, we use script theory, a concept that extends across psychological and cultural science, to assess behavioral options during interpersonal approaches. Specifically, we argue that approaches follow scripted event sequences that entail ambivalence as an essential communicative element. On the one hand, ambivalence may facilitate interpersonal approaches by maintaining and provoking situational uncertainty, so that the outcome of an action – even after several approaches and dates – remains ambiguous. On the other hand, ambivalence may increase the risk for sexual aggression or abuse, depending on the individual’s abilities, the circumstances, and the intentions of the interacting partners. Recognizing latent sequences of sexually aggressive behavior, in terms of their rigid structure and behavioral options, may thus enable individuals to use resources efficiently, avoid danger, and extricate themselves from assault situations. We conclude that interdisciplinary script knowledge about ambivalence as a core component of the seduction script may be helpful for counteracting subtly aggressive intentions and preventing sexual abuse. We discuss this with regard to the nature-nurture debate as well as phylogenetic and ontogenetic aspects of interpersonal approach behavior and its medial implementation.

## Goals

There is hardly any topic that inspires more private or public interest than interpersonal erotic approach behavior. With reference to mating, approach behavior usually encompasses seduction and attachment as well as falling or being in love. Flirting is part of the anthropological appurtenance, serving to initiate sexual and reproductive interactions (Frazier et al., 1995; Williams et al., 1999; Henningsen, 2004; Henningsen et al., 2008). Yet, interdisciplinary research has neglected either its psychobiological or its socio-cultural foundation. In the present paper on the research topic “Intercultural mental health: exceptional cognition and mating success,” we combine these two scientific aspects. In the first part of our paper, we use the script concept as a notion that extends across psychological and cultural science. We argue that seduction scripts follow predictable and stable event sequences that encompass heteronormatively rigid gender roles and sexist stereotypes. In the second part, we discuss how the reason for this rigidity may stem from a central part of the interpersonal approach, namely, ambivalence. While communicative ambivalence may facilitate sexual approaches, it also increases the risk for unwanted sexual interactions and sexual abuse. We provide insight into how interdisciplinary script knowledge may be helpful for the early detection and prevention of sexual abuse. Finally, in the third part, we examine relatively stable psychobiological as well as historically and culturally differing constituents of seduction scripts. With reference to the nature-nurture debate, we discuss future scientific and societal goals for interdisciplinary seduction research in psychology and cultural science. Conclusively, the present paper may offer a symbiotic interdisciplinary consideration of onto- and phylogenetic standards with regard to consensus in pre-sexual and sexual activities.^1^

## Mental and Cultural Scripts

### The Origin of the Collaboration

The theoretical model of the interpersonal approach script provides the scientific basis for the collaboration between cognitive neuropsychology and narratological cultural science. Schank and Abelson (1977) developed the idea that any behavior that is imagined, planned, or executed can be attributed to stable and rigid event sequences. Specifically, the conceptual representations of event sequences – or activities – in long-term memory, such as “brushing one’s teeth” or “going to the dentist” have been termed *mental scripts*. Technically, they are mentally structured along a rigid temporal dimension from the beginning of an activity until its end (Grafman, 1995; Rosen et al., 2003). Scripts entail stereotypical and situationally bound expectations and goal hierarchies, which reinforce (socially and culturally accepted) behavior (Landgraf et al., 2011, 2012). Thereby, scripts improve the predictability of events and reduce cognitive resource allocation, which represents cognitive effort, during event planning and action execution (Zacks and Tversky, 2001; Zacks and Swallow, 2007).^2^ This means, firstly, that mental scripts are determined by individual predispositions, e.g., emotional and cognitive capacities. Secondly, social and cultural factors play an important role in script formation. According to the “social cognitive theory of mass communication” (Bandura, 2001), behavioral and emotional knowledge, competences, and actions rely on both personal observation and experience, as well as on the effects of media, particularly mass media and symbolic forms (Lukesch, 2002). Due to their relative openness, mental scripts may create different demands for the participating individuals. During script execution, one’s actions and predictions of others’ reactions may be adapted and corrected in flight. Although scripts offer a plentitude of individual behavioral options, these options are simultaneously limited by the complementary, concordant, or discordant objectives of other script participants.

Importantly, similar script structures may exist in both mental representations and cultural scripts, such as narratives, novels, or movies (Fludernik, 2000; Zerweck, 2002). The cultural script codes socially shared values and knowledge, providing behavioral options. That is, narratives of all kinds provide the bases and backgrounds for behavioral proficiency, including its meanings and interpretations (Swidler, 1980). Behavior is “experienced” through media, such as movies or audio books. Moreover, these experiences, including listening, seeing, and other modalities, allow behavioral and event sequence learning, where esthetic options for empathic identification with others are of utmost importance (von Treskow, 2015). In addition, daydreaming allows people to repeat and elaborate on imagined events (Lukesch, 2002). Interestingly, these experienced actions are integrated into existing cognitive structures, which themselves determine the quality of the experience (Rosengren and Windahl, 1989; Bandura, 2001; Zacks and Tversky, 2001; Rosen et al., 2003).

Thus, real (e.g., natural) and fictitious (e.g., constructed) events help people acquire knowledge about approach behavior and its adequate contextual implementation. In order to acquire procedural and declarative knowledge about, e.g., how to flirt in a socially acceptable manner, examples are taken from reality, as well as from narratives, movies, novels, etc. Television viewers, e.g., have a rough idea about how a cinematic approach between a man and a woman takes place. Moviemakers overtly exploit this mechanism: due to the conventionalization of standardized action sequences, filmic tricks are used to purposefully evoke emotional states, e.g., surprise, anger, or despair. Given these script characteristics, we argue that disentangling behavior in general and the realization of interpersonal approach behavior specifically would benefit prominently from a psychological, cognitive, and neurobiological perspective paired with cultural science research, such as narratology, gender studies, and media science.

### The Need for Interdisciplinary Research with Regard to Approach Behavior

The Sexual Strategy Theory (SST) developed by Buss and Schmitt (1993) is of considerable interest in the context of psychological and cultural norms for seduction and interpersonal approach behavior. The SST suggests that human mating strategies have evolved to serve gender- and long/short-term-specific goals. Contextual problems are solved adaptively with regard to sexual accessibility, fertility assessment, commitment seeking, immediate resource extraction, paternity certainty, mate value assessment, and the willingness to engage in parental investment. While their work has been widely noted, it has also triggered a broad and controversial debate. In fact, the weaknesses of the SST (Fausto-Sterling, 2000; Contratto, 2002; Pedersen et al., 2011; Smiler, 2011) led Buss and Schmitt to stress the limits of their theory and the nuances of its preconditions and conclusions (Buss and Schmitt, 2011). Quite polemically and from their perspective as evolutionary psychologists, they emphasized that they do not regard gender-typical behavior as ahistoric, but instead as flexible and context-dependent (Buss and Schmitt, 2011; Smith and Konik, 2011).

This view has also been adopted with regard to sexual scripts. For example, the cultural script of sexual approaches not only influences interactions between persons, e.g., the interpersonal script. In fact, it may also affect individual preferences and needs, e.g., the intrapsychic script (Simon and Gagnon, 1986), which reciprocally may effectuate changes in the interpersonal and cultural scripts, as well. Methodologically, in culture, literature, and media science, scholars use the terms “scenario,” “narrative,” “action patterns,” or “dramaturgy” (Rumelhart, 1977; Fludernik, 1996; Eder, 1999; Hickethier, 2012; Lahn and Meister, 2013). These may be imprecise as parallel terms. Yet, they offer interdisciplinary points of contact. More currently, the script concept by Schank and Abelson has been integrated into narratology with regard to intertextual patterns (Baßler, 2005), “worldmaking” in literature and art (Fludernik, 1996; Grabes, 2010), as well as text and information processing and movie reception (Fludernik, 1996, 2000; Zerweck, 2002; Baßler, 2005; Grabes, 2010; Herman, 2013). In psychology, the relations between sexual approaches, gender codes, and mechanisms of sexualized violence have been intensively investigated via script analyses (Rose and Frieze, 1993; Klinkenberg and Rose, 1994; Frith and Kitzinger, 2001; Kim et al., 2007; Krahe et al., 2007a,b; Kray and Locke, 2008; Eaton and Rose, 2011, 2012). Nevertheless, there have not been systematic explorations that relate psychological and cultural scripts of seductive behavior to each other.

### Heteronormativity in Sexual Scripts

Heterosexual normativity appears to flood societal norms and reinforces asymmetric, that is, sexist mating strategies and expectations. In their impressive generation-spanning work, Gagnon and Simon (1987) explicate a typical temporal order for the “normative” heterosexual script in Western cultural regions. On the one hand, men are societally (and sexually) rewarded for using assertive sexual strategies toward women, for speaking openly about their own sexual desires, and for initiating or provoking – within delicately set limits – sexual behavior (Frisby et al., 2011). Moreover, since the 19th century, male sexuality has been regarded as more powerful, even more aggressive, and associated with greater subjectively perceived sexual arousal than female sexuality (Bourdieu, 1998; Wrede, 2000; Lautmann, 2002; Chivers et al., 2010). Thus, during a flirtatious encounter, male behavior is expected to be more active, especially with regard to more visible actions. On the other hand, for almost two centuries, female sexuality has been regarded as weak and passive. Women are societally rewarded for using passive, less visible, reactive, encouraging, and (from a male point of view) “alluring” strategies to win men’s affections. They objectify themselves and are objectified, and they set sexual limits (Kim et al., 2007). Whether or not these stereotypes implement gender roles is part of an international discourse. In fact, the reason for why approach scripts remain highly traditionally gender-typed may be their communicative and informative value (Eaton and Rose, 2011), especially when potential couples are getting to know each other on first dates (Afifi and Lucas, 2008).

Flirting guidebooks and Internet pages overwhelmingly demonstrate the prevalence of heteronormativity in seduction and sexual scripts (Deißler, 2012; Escaravage and Hefez, 2012). Heteronormative flirting dominates interpersonal approaches in Western societies, even though gender roles may underlie some changes (Osullivan and Byers, 1993). In fact, approach behavior in accordance with the heteronormative flirting secures and may even increase mating success (Hall and Canterberry, 2011; Beaussart et al., 2012). How and which behavioral options are actually culturally established and passed on to the next generation remain the topics of debates. Kitayama and Uskul (2011, p. 425), e.g., generously argue that cultural values are vertically passed on to the next generation because they require “an assortment of supportive beliefs and emotional conditionings.” Cultural practices, the authors state further, may be transmitted horizontally, mainly by means of adaptation. Social prestige and the urge for high status may lead to equivalent rapid practice adaptation across great distances. With regard to sexual and approach scripts, this means that these actions may be culturally mediated and simultaneously adapted through personal experience. The underlying psychological and cultural factors affect the individual at different levels and sometimes for long periods of time. Accordingly, the bases of sexual normativity may be rooted in individual circumstances, societal structures, as well as in culturally mediated value systems, including goals and their interpretations.

Differences in seduction and approach behavior between men and women cannot be accounted for only by gender, yet. For example, even though sexual consensus might seem to be culturally more closely related to affection and attachment in women than in men (Byers, 1996; Morr Serewicz and Gale, 2008; Eaton and Rose, 2011; Frisby et al., 2011; Lamont, 2015; Landgraf et al., unpublished), this does not provide proof of an etiological relationship. Further, whether or not women, “by nature,” consent to or initiate sexual activity even in the absence of sexual arousal is hard to determine. The same may hold true for the question of whether women, “by nature,” are sexually more conservative and more monogamous than men (Smiler, 2011). Overall, some kinds of behavior – such as overly aggressive flirting in men and promiscuity in women – are considered “wrong,” “non-normative,” “undesirable,” and are hence morally sanctioned in Western modern societies. Nevertheless, it is certain that the heterosexual normative script for sexual activity and approach behavior defines and demands a visibly initiating active role for men and a reserved, more careful, selective, and sexual-conduct-limiting role for women.

So, is this for good? Don’t we live in a society that promotes the ideal of equality between men and women? Why do these widespread asymmetries regarding sexual conduct persist, specifically with regard to individual goals, subjective experience, and pre-sexual behavior? Given the unequal social evaluation of sexual conduct for girls or women and boys or men, these mechanisms hold true, even though we (want to) live in a society where conservative traditions, stereotypes, and inequalities are about to disappear and even though individuals hold self-concepts of equality, freedom, and autonomy. What exactly then is it that perpetuates these asymmetries and defines asymmetries in flirting situations? What are their roles in modern and post-modern societies? In the following section, first, we focus on the function and implementation of seduction asymmetries. Second, we discuss how these asymmetries may promote sexual aggression and how they can be used for individual protection.

## Seduction Asymmetries – Uncertainty and Ambivalence

### What Is It All Good For?

Seduction asymmetries serve specific psychological and cultural functions. One of the most central aspects is a phase we call “uncertainty phase.” Its relevance during interpersonal approaches cannot be overestimated. Specifically, and we introduce this definition here, this uncertainty phase spans the period from the first detection of mutual interest until the last indication of whether a sexual interaction will occur or not. Therein, transitions from pre-sexual activity to sexual (bodily) interaction may occur only under certain circumstances. Bluntly approaching a flirting partner physically may be problematic because playful flirting suddenly becomes “more serious.” Flirting partners appear to voluntarily adhere to social norms in order to ensure they will both feel comfortable or safe in the approach situation. However, uncertainty actually needs to be evoked and maintained in order to avoid unpleasant or awkward or even dangerous situations. Therefore, ambivalent behavior impacts the situation and its participants like no other communicative and interactive element.

Cultural products celebrate many facets of this issue. The media frequently present the transition to bodily sexual interactions as delicate and with difficulty. In fact, movie scenes that present situations leading to physical closeness or rejection are of central significance to the plots of movies and their success. A famous example of the centrality of uncertainty within the context of sexual approach behavior is the fountain scene in “La dolce vita” (1960) with Marcello (Marcello Mastroianni) and Sylvia (Anita Ekberg): in this scene, time itself is extremely decelerated. The camera work and the setup suggest the erotic closeness of Sylvia and Marcello. She lures him in the form of a “bathing Venus.” He bends his body toward her, suggesting a longing and yearning. Then he follows her into the fountain. They are now part of a separate space where he tries to approach her. In fact, he chases her. This long phase of ambivalence (will he catch her or not) appears to climax into a love scene under the waterfalls of the Trevi fountain. When Marcello finally approaches her, Sylvia closes her eyes and turns toward him in the expectation of a kiss. However, he does *not* kiss her. The final decision, the ambivalence in whether the resolution of the scene will be sexual or non-sexual, changes toward the non-sexual. Marcello takes Sylvia’s hand, they leave the fountain, and he accompanies her to her hotel. The interpretation of the entire action sequence changes, and eventually the scene appears to be an innocent instance of flirting that did not explicitly lead to a sexual-physical interaction. While, technically, the sequence created erotic tension and desire, the plot changed abruptly, yet nothing was missing because the whole scene was ambivalent from the beginning.

This scene gained international popularity due to not only the skill of the director, the sophistication of the artistry, the actors, the high symbolic charge (water, moonlight, etc.), the setting, the time management, or the camera’s points-of-view. Instead, the interest of the spectators, the desire for relief, and the urge to be voyeurs specifically associated with this kind of scene played significant roles, as well. In fact, in the media and scenes like this, heteronormative elements are presented incessantly, suggesting that these elements may be vital constituents of a “successful” interpersonal approach.

Instead of mutual familiarization, seduction and sexual scripts may entail a high level of ambivalence and uncertainty in order to serve specific functions (Afifi and Lucas, 2008). That is, when ambivalence is high, uncertainty about the outcome of the interpersonal approach is high. This includes evaluating one’s own and others’ behavior, ambiguous verbal and non-verbal communication, as well as the relative openness of the outcome of the sexual approach. This may represent the fact that flirting partners may want to enhance the predicted outcome value of the interaction. By culturally coded communication, information about, e.g., potential mate fit or complementarity is reduced. This may secure emotional stability or avoid negative emotional experiences or social consequences, especially the loss of face or social privileges (Holmstrom and Burgess, 1980). Thus, ambivalence can be used strategically to maintain the interests of the flirting partners and to keep them safe from unpleasant events, including rejection or aggression. While situational ambivalence is avoided (e.g., by adhering to social standards in flirting), content ambivalence may be high (e.g., by introducing sexual content to the conversation). For example, circumstances may provide a security network made of conventional action patterns, places, timely predictions, words, and double meanings (Schank, 1975; Simon and Gagnon, 1986; Gagnon and Simon, 1987). Consequently, no matter how indirect and ambivalent the communication between the two participants, the conventional frame remains secure and stable.

While ambivalence prevails as a core element of approach behavior, uncertainty may also be reduced for specific purposes and within specific contexts. The necessity to reduce uncertainty may stem from individual differences in the tolerance of uncertainty. How comfortable an individual may feel during the uncertainty phase depends on the individual’s ability, e.g., self-confidence or experience. In other words, the capacity to endure uncertainty may also determine the degree to which ambivalence is actually present in the approach situation. On the one hand, introducing high levels of ambivalence in conversations may help in overcoming cultural sanctions on sexual behavior and increase the probability of “successful” approaches. However, on the other hand, the degree of uncertainty may also surpass an individual’s capacity. Flirting partners may not be able to tolerate a certain amount of uncertainty and, thus, they may exit the flirting situation. Since heteronormative seduction scripts can be used to reduce the amount of, e.g., uncertainty in specific situations, individuals may routinely adhere to these scripts. In fact, this may not only decrease uncertainty for participating but also for non-participating individuals (e.g., nightclubs, dates, set phrases such as “May I buy you a drink?”). However, since adhering to seduction scripts may also lead to a perpetuation of gender stereotypes (e.g., active man, passive woman), uncertainty reduction may be only reluctantly implemented during interpersonal approaches.

Finally, assigning meaning to and deducing meaning from ambivalent actions may be important factors for reducing uncertainty and increasing the number of behavioral options during interpersonal approaches. According to the four-level theory of communicating suggested by the theory of communication (Schulz von Thun, 1981), information can be perceived as mainly (a) factual, (b) a self statement, (c) a relationship indicator, or (d) an appeal. Thus, interpreting ambivalent behavior during an interpersonal approach entails knowledge about what can be expected from the current male or female flirting partner. The same is true for context-adequate behavior. Violations of expectations, e.g., surprises, may lead to physical and sexual interactions. Seismographically, the Italian movie theater illustrates this. In cases where the uncertainty phase is erased and a direct transition to sexual activity is performed, social norms appear unstable, specifically with regard to role behavior. For example, “La grande bellezza” (2013) and “La dolce vita” circulate around the meaning of sexuality in a changing social context. Federico Fellini, director of “La dolce vita,” emphasizes the epoch of the “economic miracle” as well as the beginning of the sexual liberation movement. Paolo Sorrentino, director of “La grande belleza,” addresses the turn of the second millennium, which is allegedly characterized by gender equality and technology. In both cases, sexuality signals “decadence,” revolving around control, self-confirmation, and (the absence of) reproduction. Other movies, such as ”Gegen die Wand” (2004), question the normativity of gender roles in seduction scripts. Progressively, the focus is on the impact of women who initiate sexual approaches but who cannot be discredited flatly as “bad” or “easy girls.” This exemplifies that while interpersonal approaches vary according to sociopolitical standards, deviations from the heteronormative script can occur – yet rather punctually or within the scope of a short role-play.

Some questions remain regarding the function and implementation of seduction scripts, though: how much modification regarding heteronormativity and gender roles is possible? Do differences between the sexes become visible in the form of asymmetric hierarchical elements during approaches? Could more egalitarian elements replace these asymmetric ones? Until today, high content uncertainty was central for approach behavior in both male and female flirting partners. This high degree of uncertainty, in turn, intensified the use of heteronormatively male and female elements. It is possible that this reflects the fact that only the differences between the two sexes lead to successful reproduction. However, reproduction is by far not the only goal, let alone the most important goal in bodily-sexual interactions (Holmstrom and Burgess, 1980). As a biological objective, reproduction nevertheless has a fundamental function. Hence, we hypothesize that social expectations strongly reinforce innate differences between men and women and project them outwardly way before any actual bodily contact in heterosexual encounters.

### Scripted Dangers

Moreover, we hypothesize that heteronormatively scripted interpersonal approaches propagate sexual violence and aggression. During the uncertainty phase, multiple signals regarding consent and distance are transmitted. These signals oscillate from agreement to disagreement, from speed to deceleration, from closeness to distance, etc. They may be considered constructive or destructive for the progression of the interpersonal approach. Importantly, behavior outside the heteronormative script is mostly presented as inadequate in Western media and culture. That is, “wrong” behavior is discredited, preferably as non-desirable, because non-normative behavior, in terms of the normative heterosexual script, does not provide information about heteronormativity. For example, a positive and active attitude of women toward sexuality is discredited, almost as if sexuality is something women only endure or take part in after it is initiated or with respect to successful reproduction (the “good girl” script, Kim et al., 2007). Even though women may initiate sexual approaches in Western cultures, movies such as “Gegen die Wand” show the peculiarities of the self-determined sexual actions of women, which are not oriented toward reproduction but instead carry an erotic or recreational goal (e.g., Mitchell et al., 2011). Men, on the contrary, are portrayed as potential perpetrators, importunate, superficial, and not at all interested in long-term commitment (Smiler, 2011). Burlesque caricatures of male conquest fantasies and actions serve to stabilize these patterns. In line with this, techniques that are used to delay or reject advances usually refer to female normative behavior, whereas creative strategies to undermine these techniques are presented as male normative behavior. Schemes, as described above in the fountain scene in “La dolce vita,” exemplify this, even when they illustrate the opposite: Marcello is *not* very assertive, and he does *not* kiss Sylvia. Instead of considering this non-normative behavior as a valid behavioral option for Marcello, however, not taking the chance of becoming sexual with Sylvia may be interpreted as “failing,” just in the sense of heteronormative stereotypes. Even more dramatically, since male perspectives (e.g., directors, etc.) may predominantly mark medial content and styles, it is not surprising that old patriarchic norms are reflected in cultural patterns and scientific realms (see e.g., EU-project http://eige.europa.eu/content/women-and-media-project). And as mentioned above, not conforming to the modern standards of approach behavior may actually decrease reproduction success, despite the current variety in and opportunities for behavior.

Yet, the perpetuation of sexist stereotypes and gender roles is not the only risk of stable seduction scripts. Beyond the pleasure of accelerating seductions, ambivalence and uncertainty carry the risk of sexual aggression and abuse. While multiple reasons can lead to sexual assaults (Shaw, 2005), we briefly describe three prototypical causes of such assaults that are associated with the ambivalence in heteronormative script communication: implicit misinterpretation of the woman’s behavior by the man, explicit misinterpretation, and deliberate exploitation of the uncertainty phase.

In implicit misinterpretation, the man accuses the woman of behavioral inconsistencies. While she may have been flirtatious, cooperative, and complaisant seconds ago, the woman suddenly becomes snippy and reluctant. One reason for this misinterpretation is a lack of knowledge of gender roles and gender-specific behavior. Men and women share knowledge about gender identities and gender roles (Money and Ehrhardt, 1972). By means of expectation, reward, imitation, identification, and communication, girls and women follow gender schemes that emphasize kindness and conflict avoidance. Typical gender role expectations support this: women do not achieve their goals by engaging in assertive and dominant strategies. Instead, they tend to indirectly reach their goals by being polite and courteous (Frisby et al., 2011). While flirting, this means that they “wait, encourage, and see,” that is, they react to the males’ dynamic advances yet block any sexual ones. Males, however, tend to interpret female friendliness and openness more often as indicators of sexual interest than as friendliness (Clark et al., 1999). And this difference could stem from men’s and women’s different heteronormatively determined objectives, as previously discussed (Buss and Schmitt, 1993; Kim et al., 2007; Smiler, 2011, 2013).

By contrast, explicit misinterpretations emerge when gender-typical behavior during the flirting situation is used to legitimize aggressive behavior. Culturally charged rape myths stigmatize the inconsistent behavior of women as the basis for partial responsibility in a sexual assault (Eyssel and Bohner, 2011; Sussenbach et al., 2013). Token resistance exemplifies this allegedly widespread communication strategy, which is depicted in literature, theater, opera, and movies. Just to name a few examples: Stephen Frears’ movie (1988) of the novel “Liaisons Dangereuses” (1782), Milos Forman’s version “von Treskow Valmont” (1989) or the movie “French Kiss” (1995) all accentuate yes-no-sequences. The assumption that “no” does not actually mean “no” can also be found in attempts to legitimize sexual abuse (Sick, 1995; Kieler, 2003). In interpersonal interactions, however, consent and refusals can be clearly defined and identified (Kitzinger and Frith, 1999), even if refusals may be more complex and harder to recognize that consent. Consent, on the one hand, includes a simple affirmation (e.g., “Should we get a cup of coffee? – Yes, of course!”) and does *not* require delays. On the other hand, a refusal might actually entail a short delay with a short preface (e.g., “Well, …”), palliative elements (“umm,” “err,” etc.), and a qualifying account in the form of an explanation, justification, or excuse (“I don’t have time right now but maybe later!”). Compliments and reaffirming elements may be added to ensure that one appreciates the offer and does not sound impolite (“Thank you for the offer”). Men and women show elaborated abilities to recognize culturally coded refusals (Kitzinger and Frith, 1999). In addition, girls and women learn to avoid offending their communication partners. By hiding sexual disinterest, females may avoid losing male investments or they may prevent male resentment, which could result in the perpetration of violence (Byers, 1996; Clark et al., 2009; Landgraf and von Treskow, unpublished). Hence, men experience a refusal late in an interaction as more contradictory than women, who instead see friendliness and openness rather as behavioral patterns that demonstrate social adaptation (Clark et al., 1999). Research is needed to investigate whether or not these results also hold true in other cultures (Germany, France, Italy). Different interpretations of kindness, however, reveal that gender-specific expectations in flirting and approach behavior direct and increase the risk of misunderstandings. Therefore, we hypothesize that it may actually be more useful and beneficial for women to remain in the ambivalence phase for longer. Women may avoid the danger of sexual aggression and simultaneously keep their resource options open by using this strategy. Importantly, though, sexual abuse cannot be prevented by asking women to “just say NO!” Instead, heteronormative script options for the different communication patterns of men and women need to change in order to prevent miscommunication and possibly sexual assaults.

Finally, the deliberate exploitation of the uncertainty phase occurs, for example, when someone attempts to perpetrate coercive physical or sexual contact by quickly establishing familiarity between the participants. This strategy may entail components of the yes-no theme and gender-specific misinterpretations of the situation. The processuality of approach behavior may indicate psychological and cultural factors that determine this danger in flirting situations. The mass media extensively disseminates this processuality as behavioral options during pre-sexual situations, transitions toward sexual interactions, and sexual abuse. However, what is portrayed as “mutual consent” in fact often reflects stereotypical gender patterns: male execution of pressure during approach behavior, on the one hand, and women enacting sexual reactivity on the other hand.

How then can we use cultural-mental script options to avoid or to ensure early detection of sexually aggressive situations? We argue that cultural-psychological script options have been widely neglected in the literature. Since script methodology is present in both research fields (psychology and cultural science), future qualitative and quantitative research may be based on script analyses to investigate how script knowledge can be used to identify preference-based sexual aggression. Moreover, script-based behavioral predictions rely on multimodal input (Cattaneo and Vecchi, 2011). If a prediction fails at a specific point in time, the human prediction system assumes an event boundary within the script (Newtson, 1973; Newtson et al., 1987). This implies that script predictions are constantly monitored according to perceptual input, which, in turn, may actually alter the script (Zacks and Tversky, 2001; Zacks et al., 2001, 2007; Zacks and Swallow, 2007). In addition, according to the embodied theories of cognition, event characteristics may also be used as predictors of behavioral options (Barsalou, 1999, 2003; Glenberg and Kaschak, 2002; Nuthmann and van der Meer, 2005). On the one hand, perceptual event characteristics, e.g., *imageability* (the ease with which the event can be imagined) and *complexity* (the ease with which the event can be performed), are more closely related to script experience and exposure. On the other hand, indicators of planned and goal-oriented behavior, so-called amodal event characteristics, e.g., *centrality* (Is the script possible without the event?) and *distinctiveness* (How likely is the event part of another script?), are more closely related to goal-directed behavior and script preference. When the everyday frequency of an event increases, individuals rely less on perceptual (imageability) and more on amodal (centrality) characteristics (Raisig et al., 2009). Thus, event representations influence the availability of perceptual activity information, i.e., its mental reenactment (Hard et al., 2006).

With regard to the seduction script, in situations designated as a rendezvous or a date and even in spontaneous flirting situations, this implies that individuals may be able to activate and use scripted behavior including role expectations, stereotypes, and the temporal orders of events for the early detection of danger (Rose and Frieze, 1993; Klinkenberg and Rose, 1994; Moore, 2002; Eaton and Rose, 2012). In other words, more behavioral options may be available within a shorter period of time (Landgraf et al., 2012). Since it is unclear how individual preferences influence behavioral options during pre-sexual approaches and sexual activity, investigations with regard to embodied cognition may be of utmost importance. In addition, as possible candidates for the early detection of sexual aggression, future studies should investigate the mechanisms that underlie the reduction of uncertainty, how uncertainty reduction affects gender role rigidity, and which kinds of behavioral options may decrease flirting failures and increase mutual flirting satisfaction. Here, our assumption is that individually preferred and medially popular scripts may be accessed faster and with greater detail than non-preferred ones. Finally, sensitive scientists in psychology and the cultural sciences should take into consideration how the sole act of formulating research questions and answers may already propagate traditional male and female stereotypes.

## Aspects of Nature and Nurture in Seduction Scripts

### Frontiers between the Disciplines

So far, we have examined some of the mechanisms of seduction from a culture-psychological perspective. We have seen that scripts are a methodology known to both fields, that is, as mental representations of actions or in movies and narratives. Both disciplines also confirm that seduction scripts entail a large number of heteronormative elements, such as initiating men and limiting women. While ambivalence and uncertainty are a central part of the seduction and sexual process, these elements may also build the basis for sexual aggression and abuse especially against women. In the last part of this paper, we apply the debate about the influence of psychobiologically stable and acquired cultural factors to the nature of seduction. At its core, this refers to the nature-nurture debate; the question of what primarily defines human interests and behavior – especially for those who would like to find a single answer that fits. This matter has been addressed, *inter alia*, in the United States and Europe for quite some time. Boiling it down into a few sparse lines of text is basically impossible – accordingly, the following remarks are intended as a summary that comes with no guarantee that its view is balanced. Below, we will briefly sketch how psychological research on cognition and gender studies in the cultural sciences – both of which are very wide-ranging disciplines – have reacted to each other in the context of this discussion and how either side has made a contribution to defining the nexus between the physical and the cultural. Thus, while an extensive nature-nurture debate is beyond the scope of this article, we touch upon this topic by focusing on arguments that are relevant to interpersonal sexual approach behavior.

On the one hand, psychological research tends to underestimate the role of culture, and especially the media in shaping and planning human behavior (Esposito et al., 2011). Causal explanations of natural phenomena have been attributed to restricted experimental settings in which confounding variables are either held constant or are not considered at all (Feger and Bredenkamp, 1983). Inadvertently, the psychobiological sciences overestimate the role of bodily dispositions and underestimate the role of historical attributions in the identification of gender roles. Ideological presuppositions, stemming from historical changes at the end of the Early Modern Age and over the course of the 19th century, significantly determined sexual strategies. For example, in the Early Modern Age until well into the 18th century, women were deemed significantly more libidinous and sexually more active than men. Physical aspects and thus, sexuality, were subject to the rules of self-control, age restriction, and a fixation on reproduction to a much lesser degree than today. Further, the new values of the 19th century, along with the new stigmatizations and norms made in terms of morals, work ethics, and capacity meant that men were regarded as active, aggressive, and driven by instinct. Women, however, were defined as passive, predestined to dedicating themselves fully to a given task, with a tendency to frigidity or to sexual indifference. In other words, they were portrayed as being more interested in children than in men. Since the 19th century, the general view has included gender stereotypes, such as that it is easier for women than men to exercise sexual restraint (Wrede, 2000) or that female defenses against sexuality are biological in origin (Berna-Simons, 1984). While clearly, these attributions have waxed and waned over time, they appear so deeply rooted that even academic (psychobiological) studies regard them as mechanisms that guide sexual conduct. While Western industrialization standards and the acquisition of culture-specific knowledge is important (Chivers et al., 2010), the historical development of values and standards that have been dominant since the 19th century has largely gone unaddressed. These values and standards, however, impact the physical experience of sexuality and the self-perceptions of both sexes. Nevertheless, placing the focus strictly on physical and “strategic” circumstances (strategic in the sense of reproduction or whatever is believed may be derived from it as “natural”), the historicity of sexuality, socialization, and ideological development have been neglected by the biomedical sciences.

Since the “naturalistic” approach entails the risks of neglecting historical, social, and cultural factors (Wrede, 2000), it is surprising that the insights gained in this discipline in how psychological scholars of the late 20th century have been reproducing the standards and values of the 19th century with respect to their ideational structures (i.e., men as active players vs. the passivity of women; emphasis on performance; self-control and suppression of spontaneity; postponement and renunciation of gratification; utilitarian thinking; self-optimization) are hardly given consideration in the psychobiological literature. Currently, psychological sexology is dedicating itself to psycho-physiological connections (Beier et al., 2005; Spape and Hommel, 2008; Chivers et al., 2010; Chivers and Timmers, 2012). In acknowledging cultural factors as a reason for gender differences, evolutionary psychology, the biomedical sciences, and the humanities could align themselves and profit from each other (Chivers et al., 2010; Kitayama and Uskul, 2011). For example, psychological research, specifically in cognitive and cultural psychology, approaches the positions of cultural science to a much greater degree, for example, where it studies the self-objectification of women (Kim et al., 2007) or the influence of gender stereotypes, gender roles, ideologies, and virtual reality on decisions made by women (Rudman and Heppen, 2003). However, these two studies failed to investigate physical factors and did not discuss the potential nexus between role models and physical-sexual determinants. Nonetheless, it bears noting that these latter scientific approaches do demonstrate the potential for interdisciplinary cooperation between the disciplines of neuropsychology, evolutionary psychology, and cognitive psychology on the one hand and scholars of gender studies as pursued in the cultural sciences on the other.

On the other hand, the cultural sciences, and specifically the disciplines of social and cultural gender studies, are often critical of psychobiological theories and findings (Longino, 1990; Gildemeister, 1992; Connell, 1995; Horlacher, 2010; Meuser, 2010; Gildemeister and Hericks, 2012). Here, scientists have failed to integrate scientific evidence from empirical investigations, conceptual theoretical models, and preconditions of behavior. Instead, the argumentation nowadays often appears fundamentally constructivistic. In early discussions of sex/gender, the body was in a less problematic way the starting point and was seen as leading to positive or discriminatory social codes and symbolizations (Stephan, 2006). From this point of view, the body and its organs were regarded as neutral. Afterward, research demonstrated that bodily experiences change over time, specifically, over centuries (Jacobus et al., 1990). Moreover, cultural scientists showed that in symbolizations (e.g., discourse, speech, images, and representations of all kinds), meaning was permanently being derived from the physical, that is, where sexuality and reproduction were concerned, derived from the concepts of “receiving” and “giving.” Since the 19th century, a plethora of studies have shown that the body’s reproductive function has led to a compulsive fixation on heterosexuality and certain practices (Sigusch, 1981/1988; Connell, 1995; Wrede, 2000). However, in some instances, the discussion even went so far as to almost marginalize the etiological role of the body. Here, the constructivist view argues that social meanings and interpretations pervade (sexual) self-perceptions in such a way that the objectivity of the body may be accessible only within a cultural system. In addition, in this argumentation the body might/does not have a reality of its own (Maihofer, 1995; Stephan, 2006). This line of argument removed the body from the reaches of theory to such an extent that there were no longer any ties between biological sex and social gender (Maihofer, 1995). Moreover, some positions deny even the actual existence of any biological sex (Butler, 1990).

### Commonalities and Approximations

Yet, there is some light at the end of the tunnel. While mutual reservations and criticisms reflect the difficulties of the two realms in undertaking conjoint investigations (Mathieu, 1973; Kessler and McKenna, 1978; Hubbard et al., 1979; Fox Keller, 1985; Hubbard and Birke, 1995; Héritier, 1996; Brown Travis, 2003; Liesen, 2011; Deuber-Mankowsky, 2013; Eagly and Wood, 2013), this may be explained to a significant degree by a lack of knowledge and reflection (on contextual and methodological commonalities), and, hopefully, also by a mere lack of dialog. As mentioned above, interdisciplinary theories on sexual strategies have attempted to combine approaches from sexual selection, evolutionary psychology, and endocrinology. One conspicuous aspect is that recently, both the general mode of discussion and the latest combinations of methods and theories have become more conciliatory in nature (e.g., Geary, 2009; Ellis, 2011; Pedersen et al., 2011; Tate, 2013). The tone, the presentation of evidence, and the line of arguments have all undergone noticeable change. Thus, the objectives of female and male individuals are no longer just called “reproduction” and “childrearing”; instead, it is postulated that men and women must find solutions conjointly (Geary, 2009; Hannagan, 2011), e.g., create “reproductive alliances” (Buss and Schmitt, 1993, 2011). Linear, one-track reasoning is no longer acceptable. Instead, the focus in recent studies has been on the interaction of mechanisms, on how individuals interact, or in other words on reciprocal responses in the broadest sense of the term (Buss and Schmitt, 2011; Ellis, 2011). However, non-cultural scientific investigations have still failed to consider qualitative constructivist or cultural scientific reasoning.

Ellis (2011) suggested that in the course of human evolution the male brain has evolved to react to female preferences and that this adjustment, via the hormones of men, has become part of their genetic make-up. Accordingly, male behavior and the male self-image could be understood as a special demonstration of one’s own person as a “competent resource provisioner” (Ellis, 2011, p. 707). This is tantamount to an ideological about-face: it is not the men who choose the women; it is the women who choose their men, which is why men have adjusted to the parameters of women’s mating preferences (“women have shaped the average man into being a resource provisioner,” p. 718). While Ellis considers his theory to be a universal one, he also emphasizes the significance of the differences that are specific to certain cultures, the importance of learning, as well as the fact that in actual concrete situations, behavior need not absolutely comply with these requirements.

Ellis’ and other theories appear sophisticated, comprehensible, and intelligible. While they unite the extremes of neurological and behavioral research perspectives, from a constructivist point of view, Ellis’ theory may be regarded as a displacement of the argument from an evolutionary discussion, as in Buss and Schmitt’s papers, to hormonal and genetic factors. This, however, may just be a shift in the line of combat, without changing the actual positions. The underlying argument, that men and women are different from a deterministic point of view, is still maintained. Early on in the debate, Fausto-Sterling (1985, 2000) noted how endocrinology has been used to provide a basis for stereotypes without greater reflection or introspection. Therefore, research is still needed to investigate whether or not newer theories are, in fact, suited to overcome old issues regarding genetic determination and the constructed flexibility of gendered concepts.

## Conclusion

This article on the seduction script suggests that the union of two scientifically rather distant disciplines – psychology and cultural science – may contribute to a better understanding and a solution for societally relevant challenges. Importantly, these research fields are connected by how closely methodology and content of the script concept resemble each other in the two disciplines. Moreover, the theoretical collaboration presented here can already provide the following conjectures with regard to behavioral options during interpersonal sexual approach behavior.

First, sexual approach behavior follows scripted, predefined event sequences that entail psychobiological and cultural standards. As a core component, communicative ambivalence enforces heteronormative, predictable behavioral patterns, which may be used to decrease uncertainty (situational conventions, dress and conversational codes, etc.) or to increase uncertainty (sexual or attachment intentions, emotional involvement, etc.) during flirting situations.

Second, used as playful manipulations, ambivalent components of the seduction interaction may facilitate sexual aggression and abuse, depending on the capacities and objectives of individuals as well as circumstantial factors. Due to the relative rigidity of behavioral options, flirting individuals could recognize dangerous and potentially hazardous situations early. The more obviously an ambivalent situation is being provoked or maintained, the more careful (and skeptical) the interacting partner should be. Thus, aggressive intentions may be undermined by using knowledge about scripted ambivalence as a core component of seduction interactions.

Third, the present paper proposes a topic-specific interdisciplinary approach for unraveling the contribution of psychobiological predispositions as well as cultural standards for behavior. Hence, with regard to interpersonal approach and sexual behavior, we propose that future studies should investigate how collectively accepted behavioral options (e.g., gender-specific asymmetries or communicative ambivalences) are related to (probably) invariable psychobiological preconditions. More than simply enlarging our understanding of the underlying causes for the persistent perpetuation of sexist stereotypes, gender roles, and machismo, this approach may also integrate neuropsychological, forensic-biological, and socio-cultural backgrounds with regard to highly aggressive behavior and sexualized violence, especially against women.

## Author Contributions

All authors listed, have made substantial, direct and intellectual contribution to the work, and approved it for publication.

## Conflict of Interest Statement

The authors declare that the research was conducted in the absence of any commercial or financial relationships that could be construed as a potential conflict of interest.

## Footnotes

1 Some of the ideas from this paper were presented in the German journal “Blick in die Wissenschaft” in June 2015. Here, we advance and further develop our ideas in more depth.

2 For an interesting discourse-related theoretical approach that considers the roles of scripts in human conversations, please refer to Edwards (1994).
